# Molecular subtypes of urothelial carcinoma are defined by specific gene regulatory systems

**DOI:** 10.1186/s12920-015-0101-5

**Published:** 2015-05-26

**Authors:** Pontus Eriksson, Mattias Aine, Srinivas Veerla, Fredrik Liedberg, Gottfrid Sjödahl, Mattias Höglund

**Affiliations:** 1 grid.4514.40000000109302361Division of Oncology and Pathology, Department of Clinical Sciences Lund, Lund University, Lund, Skåne SE-223 81 Sweden; 2 grid.4514.40000000109302361Division of Urological Research, Department of Clinical Sciences Malmö, Lund University, Malmö, Skåne SE-205 02 Sweden

**Keywords:** Urothelial carcinoma, Bladder cancer, Transcription factor, Gene signatures, Molecular subtypes

## Abstract

**Background:**

Molecular stratification of bladder cancer has revealed gene signatures differentially expressed across tumor subtypes. While these signatures provide important insights into subtype biology, the transcriptional regulation that governs these signatures is not well characterized.

**Methods:**

In this study, we use publically available ChIP-Seq data on regulatory factor binding in order to link transcription factors to gene signatures defining molecular subtypes of urothelial carcinoma.

**Results:**

We identify PPARG and STAT3, as well as ADIRF, a novel regulator of fatty acid metabolism, as putative mediators of the SCC-like phenotype. We link the PLK1-FOXM1 axis to the rapidly proliferating Genomically Unstable and SCC-like subtypes and show that differentiation programs involving PPARG/RXRA, FOXA1/GATA3 and HOXA/HOXB are differentially expressed in UC molecular subtypes. We show that gene signatures and regulatory systems defined in urothelial carcinoma operate in breast cancer in a subtype specific manner, suggesting similarities at the gene regulatory level of these two tumor types.

**Conclusions:**

At the gene regulatory level Urobasal, Genomically Unstable and SCC-like tumors represents three fundamentally different tumor types. Urobasal tumors maintain an apparent urothelial differentiation axis composed of PPARG/RXRA, FOXA1/GATA3 and anterior HOXA and HOXB genes. Genomically Unstable and SCC-like tumors differ from Urobasal tumors by a strong increase of proliferative activity through the PLK1-FOXM1 axis operating in both subtypes. However, whereas SCC-like tumors evade urothelial differentiation by a block in differentiation through strong downregulation of PPARG/RXRA, FOXA1/GATA3, our data indicates that Genomically Unstable tumors evade differentiation in a more dynamic manner.

**Electronic supplementary material:**

The online version of this article (doi:10.1186/s12920-015-0101-5) contains supplementary material, which is available to authorized users.

## Background

Urothelial carcinoma (UC) arises from the urinary bladder epithelium that consists of three cell layers, basal cells, transiently amplifying cells and differentiated umbrella cells. Basal cells typically express KRT5 and CDH3 (P-cadherin), whereas KRT20 and uroplakins are expressed by umbrella cells. Upon injury, stromal signals induce proliferation of basal cells that ultimately form the more differentiated cell layers [1]. Key factors in urothelial development and differentiation have been extensively studied during the last decade. These studies have shown that nuclear receptors PPARG and RXRA, as well as the transcriptional regulators FOXA1, TP63 and GATA family members govern different aspects of the differentiation process [2]. Using molecular stratification we have previously described three major subtypes of urothelial carcinoma; Urobasal (A and B), Genomically Unstable and SCC-like [3, 4]. Urobasal A (UroA) tumors show papillary growth, good prognosis and frequent mutation and expression of *FGFR3*. Urobasal B (UroB) tumors are biologically and clinically progressed but molecularly similar to UroA tumors [3, 4]. Genomically Unstable (GU) tumors are undifferentiated, highly proliferative and characterized by frequent *E2F3/SOX4* amplifications, *RB1* deletions, *TP53* mutations and *ERBB2* expression [4–6]. SCC-like (SCCL) tumors show enhanced expression of basal urothelial markers *KRT5*, *EGFR* and *CDH3*, signs of keratinization and squamous differentiation. Here we use publically available ChIP-Seq data on regulatory factor binding to link transcription factors to gene signatures defining molecular subtypes of UC. We identify loss of *PPARG* and *ADIRF*, a novel regulator of fatty acid metabolism, as well as upregulation of *STAT3* expression, as putative mediators of the SCCL phenotype. We link the PLK1-FOXM1 axis to the rapidly proliferating GU and SCCL subtypes and show that differentiation programs involving PPARG/RXRA, FOXA1/GATA3 and HOXA/HOXB are differentially expressed in UC molecular subtypes. We expand on the suggested similarities between UC and breast cancer [7], and show that gene signatures and regulatory systems defined in urothelial carcinoma operate in breast cancer in a subtype specific manner suggesting similarities at the gene regulatory level of these two tumor types.

## Methods

### Experimental design

The current study is based on the Lund 308 UC tumor cohort for which gene expression data (GSE32894) and tumor molecular subtype is available [3]. Informed consent was obtained from all patients and the study was approved by the Local Ethical Committee of Lund University in accordance with the Helsinki declaration. For the present investigation, samples belonging to the “Infiltrated” subtype were excluded as the gene expression profile of this subtype is heavily compromised by infiltrating immunological cells, leaving 131 UroA, 21 UroB, 85 GU and 29 SCCL cases. Processing and subtyping of the Chungbuk UC dataset (*n* = 165) [8] was performed as described previously [3]. Gene expression datasets generated by The Cancer Genome Atlas Network (TCGA) for bladder [9] (RNASeqV2, *n* = 223) and breast [10] (Agilent 244 K, *n* = 547) were obtained through the TCGA data portal. Whole-genome transcription factor (TF) binding site data derived from chromatin immunoprecipitation sequencing (ChIP-Seq) was obtained from the ENCODE regulation super-track [11] via the UCSC Genome Browser. Promoter regions (−5000 - +1000 bp from the transcription start site (TSS)) of the longest transcript of each RefSeq gene (23 645 genes at dataset creation date 2013–01) were analyzed for TF ChIP-Seq peaks using the “GenomicRanges” package in R [12]. CISTROME ChIP-Seq data was downloaded from the Nuclear Receptor Cistrome Database [13] and mapped to promoter regions in the same way. One additional PPARG ChIP-Seq dataset was downloaded from CistromeFinder portal [14]. ChIP-Chip data for 39 nuclear receptors and cofactors in MCF-7 cells generated by Kittler *et al*. [15] was downloaded and their reported 50 kb TF-peak to gene-TSS assignments used. HOXA2 mouse ChIP-Seq data [16] was used to identify potential human HOXA2 binding promoters. Gene symbols were translated using bioDBnet [17]. Enrichment of HOX DNA motifs was analyzed with the SMART software [18] using position weight matrices from TRANSFAC Professional [19].

### Immunohistochemistry

Tissue microarrays (1.0 mm cores, 4 μm sections) were analyzed with antibodies against CCNB1 (Y106, Epitomics), EGFR (3C6, Ventana), FOXA1 (2F83, Abcam), FOXM1 (C-20, Santa Cruz), GATA3 (D13C9, Cell Signaling), phospho-HISTH3 (Ser10) (#9701, Cell signaling), KRT5 (EP1601Y, Thermo Scientific), LAMA5 (4C7, Dako), PLK1 (208G4, Cell signaling), PPARG (C26H12 Cell Signaling), RXRA (F-1, Santa Cruz), STAT3 (124H6, Cell signaling), phospho-STAT3 (Tyr705) (D3A7, Cell signaling). Cores were evaluated as blinded digitalized image files. For quantitatively staining markers (EGFR, FOXA1, GATA3, KRT5, PPARG, RXRA, STAT3) a tumor cell score (TCS) was defined as described in Sjödahl *et al*. [4]. For discrete cellular labeling (CCNB1, FOXM1, PLK1, p-STAT3), fractions of positive tumor cells was recorded. The mean tumor cell score of core pairs from the same sample was calculated. The number of cores evaluated for each marker ranged from 480 to 524. Mitotic figures were identified by the phospho-HISTH3 (Ser10) antibody and basal lamina by anti-Laminin α-5 staining. When possible, lines were drawn through the plane of the mitotic figure (in the direction of the cell division), and tangentially along the nearest basal lamina (Photoshop CS5 version 12.1), and the distance, in cell layers, to the basal membrane recorded. A total of 416 mitoses from 112 different tumors were analyzed.

### Statistical analysis

Quality Threshold Clustering (QTC) was performed with a sample jackknife correlation cut-off at 0.4, with a minimum cluster size of 15 genes. Significance Analysis of Microarrays (SAM) was performed with a FDR cut-off set at 0 in all analyses [20]. SAM and QTC gene lists are supplied in Additional file 1: Table S4. Literature derived associations between gene lists and transcriptional regulation was analyzed using GeneGo MetaCore™ (Thomson Reuters). Transcription factor binding motif enrichment was examined both on the full promoter sequence (−5000 - +1000 bp from TSS) as well as DNaseI footprint filtered sequence [21]. In-silico enrichment of ChIP-Seq binding was analyzed by two methods; a one-tailed Fisher’s exact test and a resampling based test examining both the number of bound promoters as well as the total number of identified ChIP-Seq peaks in a gene list compared to 100 000 randomly sampled gene lists of equal size. Only array probes mapping to RefSeq genes were included for each analysis.

## Results

### Coordinated downregulation of a urothelial differentiation module in the SCC-like UC subtype

**Fig. 1:**
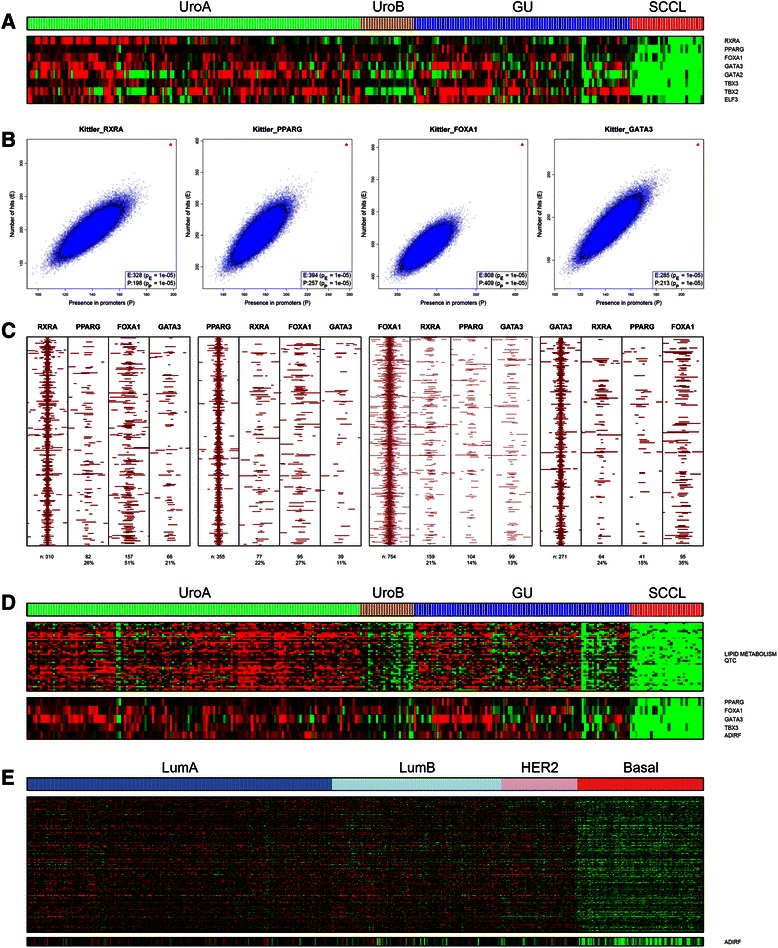
Genes downregulated in the SCC-like subtype. **a** Transcription factors strongly downregulated in the SCCL subtype included RXRA, PPARG, FOXA1, GATA3, GATA2, TBX2, TBX3 and ELF3. **b** Enrichment of RXRA, PPARG, FOXA1 and GATA3 binding in the SCCL-down genes. (red, query signature; blue, 10^5^ randomly sampled gene lists of equal size to the query signature). **c** Colocalization of RXRA, PPARG, FOXA1 and GATA3 binding events in SCCL-down genes using the Kittler data set in a ±1000 bp window centered on the first factor in each subpanel. **d** A 42 gene QTC cluster, enriched for the GO term lipid metabolism included the transcription factors PPARG, FOXA1, GATA3, TBX3 and the adipogenesis regulatory factor ADIRF. **e** Downregulated genes involved in lipid metabolism (*n* = 233) in the TCGA BC basal-like subtype

We applied SAM analysis to identify significantly downregulated genes in the SCCL subtype (SCCL-down, *n* = 829 genes). To screen for possible upstream regulatory factors we analyzed the SCCL-down gene list using GeneGO MetaCore, which revealed a regulatory association between the downregulated genes and the PPARG and RXRA transcription factors/nuclear receptors, both downregulated in the SCCL subtype. PPARG and RXRA cooperate as heterodimers and induce differentiation though the transcription factors ELF3, FOXA1 and members of the GATA transcription factor family [22, 23] (Additional file 2: Figure S1). The *PPARG*, *RXRA*, *GATA2*, *GATA3*, *FOXA1* and *ELF3* transcription factors genes were among the most downregulated in the SCCL-down list (Fig. 1a, Additional file 2: Table S2). We mapped ChIP-Seq and ChIP-Chip derived transcription factor binding sites obtained from ENCODE, CISTROME and Kittler *et al*. to all RefSeq gene promoters in order to calculate in-silico ChIP-Seq binding enrichment. The SCCL-down gene list was highly enriched for RXRA, PPARG, FOXA1 and GATA3 binding sites with p-values ranging from 10^−4^ to 10^−18^ (Additional file 2: Table S1A), corroborated by the resampling-based test (Fig. 1b). ChIP-Seq peak calls indicated binding of at least two of these factors in a majority (60 %) of SCCL-down promoters (Additional file 2: Figure S2), as well as spatial clustering of binding sites (Fig. 1c). The SCCL-down genes were also enriched for RARA and RARB binding sites. While the mRNA levels of the RAR genes did not vary between subtypes, we observed SCCL specific downregulation of ALDH1A2 and overexpression of CYP26B1, involved in synthesis and degradation of the retinoid RAR ligands, respectively (Additional file 2: Figure S3). We used bootstrap hierarchical clustering [24] to identify the SCCL subgroup (*n* = 71) of tumors in The Cancer Genome Atlas Network (TCGA) bladder cancer data (Additional file 2: Figure S4), and derived an independent list of SCCL downregulated genes. We found a significant overlap between the two SCCL-down lists (581 of 829 genes). The transcription factors *PPARG*, *FOXA1*, *GATA3*, *GATA2* and *ELF3* ranked among the top downregulated genes also in the TCGA SCCL subgroup (Additional file 2: Figure S4, and Table S2). *RXRA* was not among the significant genes as expression of *RXRA* is a characteristic of low stage tumors absent in the TCGA dataset (Fig. 1a). In-silico ChIP-Seq analysis showed strong enrichment for RXRA, PPARG, FOXA1 and GATA3 binding also at TCGA-SCCL-down gene promoters (Additional file 2: Table S1B).

### PPARG regulated lipid metabolic genes are downregulated in the SCC-like UC subtype

The SCCL-down list was highly enriched for genes annotated with “lipid metabolism” (116 of 829, *p* < 10^−24^). In-silico ChIP-Seq analysis of the promoters revealed a strong enrichment for PPARG binding (40 % bound, *p* < 10^−6^), in line with PPARG involvement in lipid metabolism [25]. Unsupervised quality threshold clustering (QTC) identified a tightly correlated cluster of genes (*n* = 42) enriched for GO term “lipid metabolic process” (*p* < 10^−5^). This cluster was strongly downregulated in the SCCL subtype (Fig. 1d) and included the transcription factors *PPARG*, *TBX3*, *FOXA1*, *GATA3* and *ADIRF* (*c10orf116*). ADIRF promotes adipocyte differentiation by acting upstream of and inducing PPARG expression [26]. This hierarchy of regulation is supported by the finding that the ADIRF promoter region does not contain any PPARG ChIP-Seq binding sites. We were unable to analyze ADIRF binding as ChIP-Seq data has not been generated for this factor. ADIRF was also the top ranking transcriptional regulator in the TCGA SCCL-down gene lists, supporting a role in urothelial carcinoma.

### A STAT3 regulated gene signature is upregulated in the SCC-like UC subtype

**Fig. 2:**
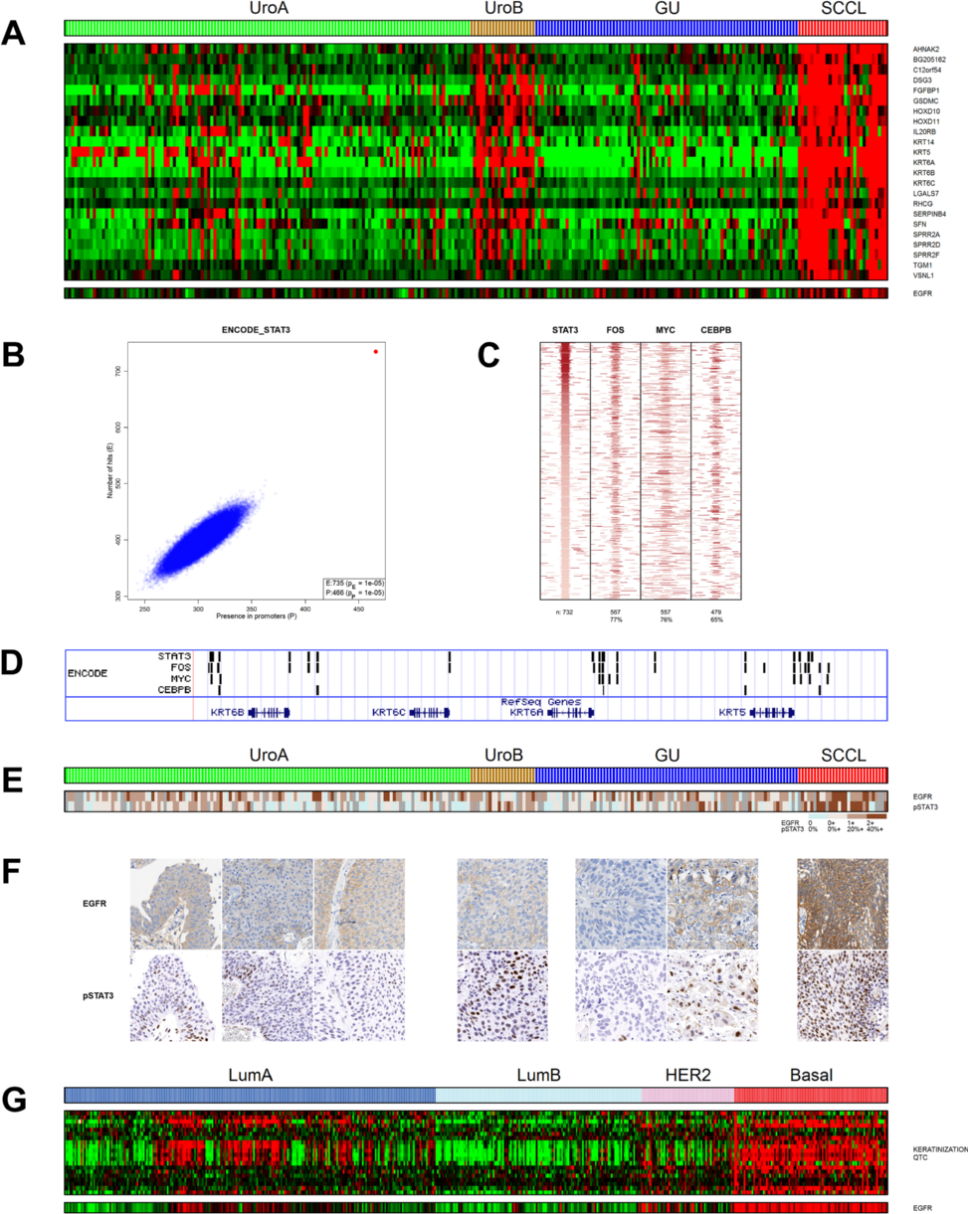
Genes upregulated in the SCC-like subtype. **a** A 23 gene QTC cluster containing multiple genes involved in keratinization including KRT5, KRT6A-C and KRT14. EGFR expression is increased in the SCCL subtype. **b** Enrichment of STAT3 binding in SCCL-up genes (red, query signature; blue, 10^5^ randomly sampled gene lists of equal size to the query signature). **c** Colocalization of FOS, MYC and CEBPB with STAT3 binding sites in the promoters of SCCL-up genes, using ENCODE ChIP-Seq data, window centered on STAT3. **d** STAT3, FOS, MYC and CEBPB binding sites in the promoters of the KRT5 and KRT6 genes. **e** Heat map indicating the EGFR and pSTAT3 IHC staining scores, respectively. **f** IHC sections stained for EGFR (top) and pSTAT3 (bottom) showing characteristic staining patterns of the bladder cancer subtypes. **g** Expression pattern of the bladder derived keratinization QTC cluster genes and EGFR in the TCGA breast cancer dataset

We identified a tightly correlated QTC cluster (“Keratinization”) upregulated in SCC-like UC that included *KRT5*, *KRT6A*, *KRT6B*, *KRT6C*, *KRT14* and *KRT16* (Fig. 2a). In-silico ChIP-Seq analysis of promoters revealed strong enrichment of STAT3 and FOS binding (72 % and 81 % bound respectively, both *p* < 10^−4^). We performed a SAM analysis to derive an expanded list of genes upregulated in SCC-like UC (SCCL-up, *n* = 1085 genes), and found strong enrichment of STAT3 binding at SCCL-up gene promoters (44 % bound, *p* < 10^−25^), corroborated by the resampling-based analysis (Fig. 2b). We also detected enrichment of FOS (57 % bound, *p* < 10^−21^), MYC (57 % bound, *p* < 10^−21^), and CEBPB (60 % bound, *p* < 10^−11^) binding at SCCL-up gene promoters. Binding peaks for these transcription factors exhibited a high degree of spatial overlap (Fig. 2c). The high density of STAT3 binding sites within *KRT5* and *KRT6* promoters links STAT3 activation with KRT5/6 expression (Fig. 2d). SCCL tumors display nuclear protein expression of phospho-STAT3 showing that STAT3 is activated in these tumors. In addition, EGFR that both activates and interacts with STAT3 [27, 28] showed increased expression in the SCCL subtype (Fig. 2e-f).

### Similarities between SCC-like UC, basal-like breast cancer and squamous cell carcinoma of the lung

**Fig. 3:**
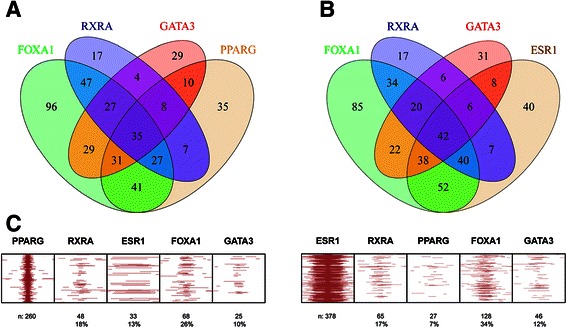
Promoter binding overlap in UC and BC downregulated genes. **a** Co-occurrence of FOXA1, RXRA, GATA3 and PPARG binding in promoter regions of the 664 genes downregulated both in the UC TCGA SCCL and the BC TCGA basal-like subtypes. Co-occurrence derived from the Kittler dataset. **b** Co-occurrence of FOXA1, RXRA, GATA3 and ESR1 binding in promoter region of the 664 genes downregulated both in the UC TCGA SCCL and the BC TCGA basal-like subtypes. Co-occurrence derived from the Kittler dataset. **c** Co-localization of binding events. ChIP-Seq peaks in the promoters of the 664 genes downregulated in both the bladder SCCL and breast basal-like subtypes, centered on PPARG binding events (left), and ESR1 (right) binding events

We extracted a breast cancer (BC) basal-like down gene signature from breast cancer TCGA data. This signature included *FOXA1*, *GATA3*, *ESR1*, as well as *ADIRF*, as high ranking genes (ranks 1, 11, 13 and 139, respectively, out of 3638 genes) (Additional file 2: Table S2). ChIP-Seq data revealed that BC basal-like downregulated gene promoters were highly enriched for RXRA, PPARG, FOXA1, GATA3, as well as for ESR1 binding (Additional file 2: Table S1C). To extend this comparison we identified 664 genes that were downregulated in SCCL UC and basal-like BC in the TCGA data sets. Binding of PPARG, RXRA and ESR1 to the respective promoters were highly significant (*p*-values 10^−4^, 10^−7^ and 10^−10^, respectively), and exhibited spatial overlaps at promoters (Fig. 3a-c). Prat *et al*. [29] highlighted similarities between basal-like BC and lung squamous cell carcinoma and defined a gene signature shared between the two cancer types. Of the 300 downregulated genes in this shared signature, *FOXA1*, *GATA3*, and *ESR1* were among the top ranking transcription factors (ranks 10, 3 and 1, respectively). These observations support downregulation of the FOXA1 and GATA3 transcription factors as important components in maintaining a basal/SCC-like phenotype. Furthermore, the breast cancer basal-like down genes were enriched for “lipid metabolic process” (*p* < 10^−5^, *n* = 233), indicating that analogous processes are inhibited in both SCCL UC and basal-like BC (Fig. 1e). Overexpression of the correlated keratinization QTC genes and EGFR was observed also in basal-like BC (Fig. 2g), further emphasizing the similarity between the respective UC and BC subtypes.

### A PLK1-FOXM1 cell cycle axis operating in the GU and SCC-like UC subtypes

**Fig. 4:**
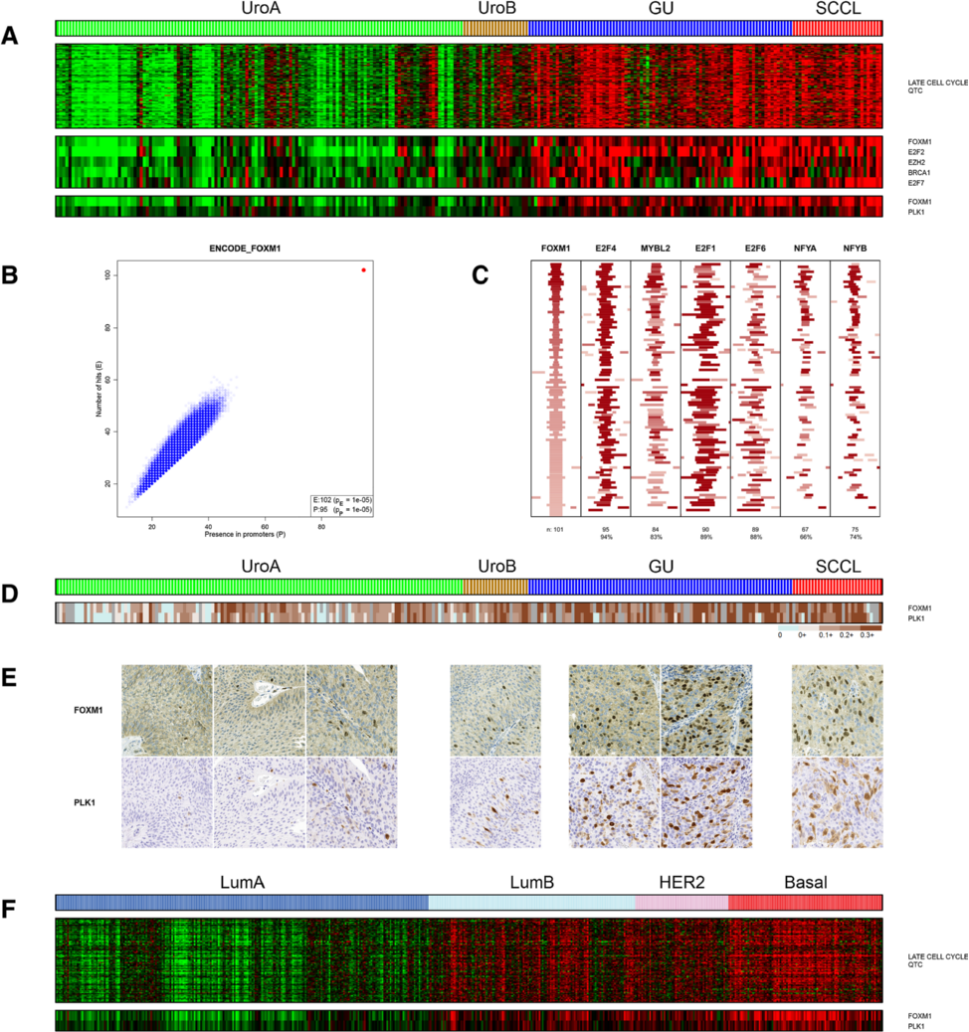
FOXM1 regulated genes in UC. **a** A QTC cluster of 129 genes linked to the late cell cycle including the transcription factors FOXM1, among others. PLK1 mRNA expression levels are indicated. **b** Enrichment of FOXM1 promoter binding in late cell cycle QTC genes (red, query signature; blue, 10^5^ randomly sampled gene lists of equal size to the query signature). **c** Transcription factors associated with cell cycle regulation and the DREAM complex colocalizes with FOXM1 binding events in the late cell cycle QTC gene promoters. **d** Heat map indicating the percentage of FOXM1 and PLK1 positive tumor nuclei in TMA cores. **e** FOXM1 (top) and PLK1 (bottom) stained sections of representative tumors across the UC subtypes. **f** The UC derived late cell cycle gene cluster in the breast cancer TCGA gene expression dataset

We identified a tightly coordinated QTC cluster (“Late Cell Cycle”) of 129 proliferation associated genes highly expressed in GU and SCCL tumors (Fig. 4a). This cluster included several transcriptional regulators, *e.g., FOXM1*, *E2F2*, *EZH2*, *E2F7* and *BRCA1* (Fig. 4a). Furthermore, GeneGO MetaCore analysis of the gene list suggested a link to transcription factors *E2F1*, *E2F2*, *E2F3*, *MYC*, *MYBL2* and *FOXM1* (all *p* < 10^−8^). ChIP-Seq analysis showed strong enrichment of DREAM complex [30] members FOXM1 (76 % bound, *p* < 10^−35^, Fig. 4b), E2F4 (*p* < 10^−35^), and MYBL2 (*p* < 10^−31^) as well as other cell cycle regulatory factors *e.g.,* E2F1 (*p* < 10^−30^), NFYA (*p* < 10^−29^), and NFYB (*p* < 10^−30^) (Additional file 2: Table S3). ChIP-Seq peaks for these factors all exhibited a high degree of spatial overlap with the FOXM1 peaks (Fig. 4c). FOXM1 is activated by a phosphorylation feedback loop mediated by PLK1 [31]. FOXM1 and PLK1 protein expression showed overlapping (IHC) nuclear labeling patterns and were highly correlated across the data (r = 0.75, *p* < 10^−15^) (Fig. 4d-e). The UC derived *FOXM1* late cell cycle signature showed coordinated expression in the BC TCGA data with Luminal A showing low and Luminal B, HER2-enriched and basal-like high expression (Fig. 4f).

### Urobasal A tumors express RXRA, PPARG, FOXA1 and GATA3 at the protein level

**Fig. 5:**
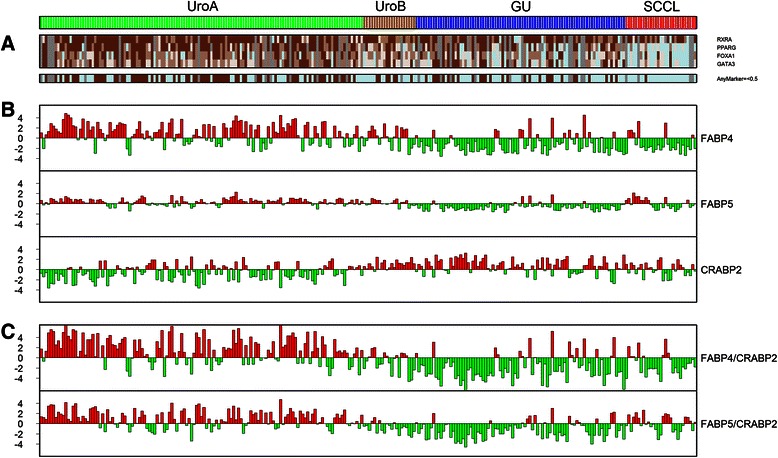
UroB, GU and SCC-like tumors lose transcription factors involved in urothelial differentiation and have altered expression of intracellular lipid-binding proteins. **a** Heat map illustrating nuclear IHC staining for transcription factors involved in urothelial differentiation. IHC scores for the individual transcription factors (top). Loss of any transcription factor (IHC score <0.5) indicated with blue (bottom). **b** FABP4, FABP5 and CRABP2 mRNA expression across UC subtypes. Red bars up, expression above median; green bars down expression below median **c** The ratio between FABP4/5 and CRABP2 mRNA levels, respectively

We explored protein expression of the differentiation factors RXRA, PPARG, FOXA1 and GATA3 using IHC (Fig. 5a and Additional file 2: Figure S5). Protein expression correlated with mRNA levels and was high in UroA, intermediate in UroB and GU and absent in SCCL. Using lack of protein expression (Tumor Cell Score < 0.5, with a range of 0–3) of at least one member of the circuit as an indication of a non-functional pathway, the full complement of the circuit was maintained to larger extent in UroA compared to the rest (*p* < 10^−6^, Fishers exact test), as well as when compared with GU only (*p* < 10^−3^) (Fig. 4a). We then investigated mRNA expression of fatty acid binding proteins (FABP4 and FABP5) and cellular retinoic acid-binding protein (CRABP2) specifically. These proteins are involved in shuttling of lipophilic ligand compounds and retinoic acid (RA) to the PPAR and RAR nuclear receptors [32]. UroA cases showed high expression of *FABP4* and *FABP5* and low expression of *CRABP2* mRNAs, whereas the reverse was observed for UroB, GU and SCCL cases (Fig. 5b). The *FABP4/5* and *CRABP2* gene expression ratios, crucial for ligand shuttling and response [33, 34], show an even stronger contrast between UroA and the remaining UroB, GU and SCCL subtypes (Fig. 5c).

### Urobasal A tumors express anterior HOXA and HOXB genes

**Fig. 6:**
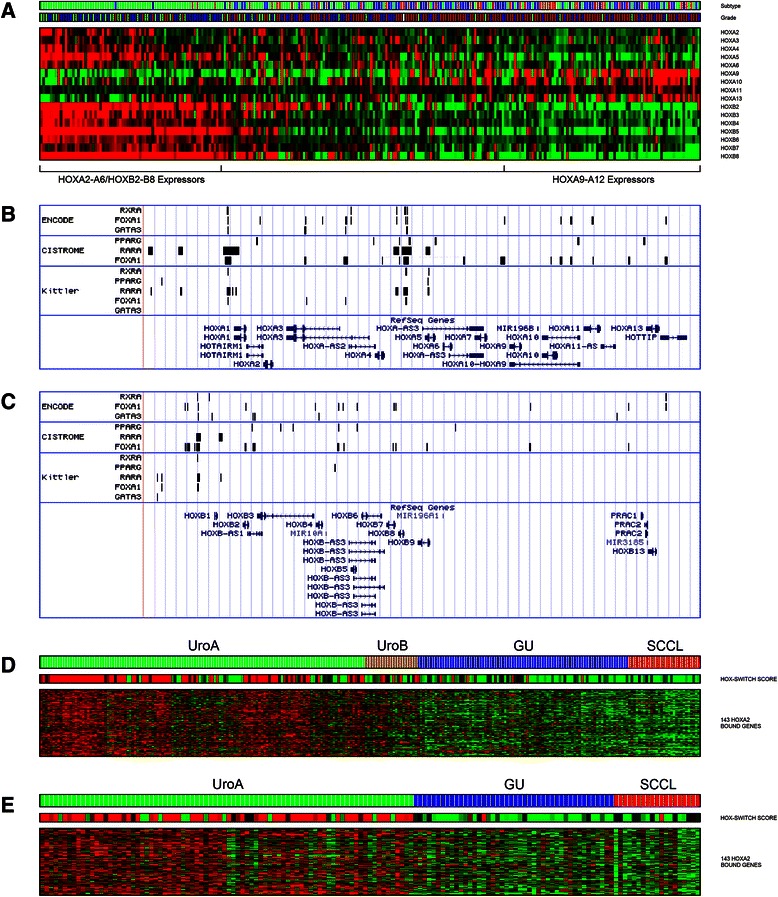
A HOXA/HOXB switch in urothelial carcinomas. **a** Expression of HOXA and HOXB genes. Cases arranged according to HOX-switch score. The HOXA2-A6/HOXB2-B8 expressing group consists mainly of Urobasal A tumors and is enriched for grade G1 tumors. Anterior HOXA **b** and HOXB **c** gene clusters show frequent binding sites for RXRA, PPARG, FOXA1, GATA3 and for RARA. **d** The expression of HOXA2 binding genes across the Lund dataset. Bar above heat map indicates HOX-switch score (red, high score; green, low score). **e** The expression of HOXA2 binding genes across the Chungbuk cohort. Bar above heat map indicates HOX-switch score (red, high score; green, low score)

We have previously documented a differentiation-related switch in DNA methylation states between anterior (HOXA1-7) and posterior (HOXA9-13) HOXA genes [35]. To explore the differential HOX-gene expression in the context of UC molecular subtypes, we assigned each tumor a HOX-score based on the balance of anterior *HOXA* and *HOXB* versus posterior *HOXA* gene expression (Additional file 2: Figure S6). Rank-ordering tumors by HOX-score clearly separated tumors expressing anterior *HOXA* and *HOXB* genes (high HOX-scores) from tumors expressing only posterior *HOXA* (low HOX-scores) genes (Fig. 6a). Tumors with high HOX-scores were of low grade and strongly enriched for the UroA subtype (Fig. 6a). Similar patterns of differential HOXA/HOXB expression were not observed in the TCGA BC data (Additional file 2: Figure S7A). ChIP-Seq analysis showed frequent RXRA, PPARG, FOXA1, GATA3 and RARA binding within the anterior *HOXA* and *HOXB* locus (Fig. 6b-c). As we were unable to find ChIP-Chip or ChIP-Seq data for human anterior HOXA genes, we made use of HOXA2 ChIP-Seq data generated in mouse [16], and translated the list of mouse target genes to human orthologs. Mapping of TRANSFAC transcription factor binding motifs revealed substantial enrichment of HOX motifs across the human ortholog promoters. We used SAM to derive a list of genes upregulated in tumors with a high HOX-score and quantified the overlap with the translated HOXA2 target gene list. Of 672 upregulated genes, 143 (23 %) were putative HOXA2 targets in mouse, a significant enrichment (*p* < 10^−11^, Additional file 1: Table S4). The expression pattern of human ortholog HOXA2 target genes mirrored the HOX-score with high expression levels in UroA cases, intermediate levels in UroB and low expression in GU and SCCL cases (Fig. 6d). The HOX-score and HOXA2 gene signature expression patterns were recapitulated in an independent UC dataset (Fig. 6e, Additional file 2: Figure S7B). HOXA2 target genes were enriched for GO terms related to development *e.g.,* cell differentiation (*p* < 10^−8^), epithelium development (*p* < 10^−6^), and urogenital system development (*p* < 10^−4^), and included the genes *TP63, GATA3, TBX3, ELF3, BMP7, TGFBR2, TGFBR3, SMAD3* and *SMAD6*, all with documented roles in differentiation and developmental processes.

### Urobasal A tumors show basal membrane associated cell divisions

**Fig. 7:**
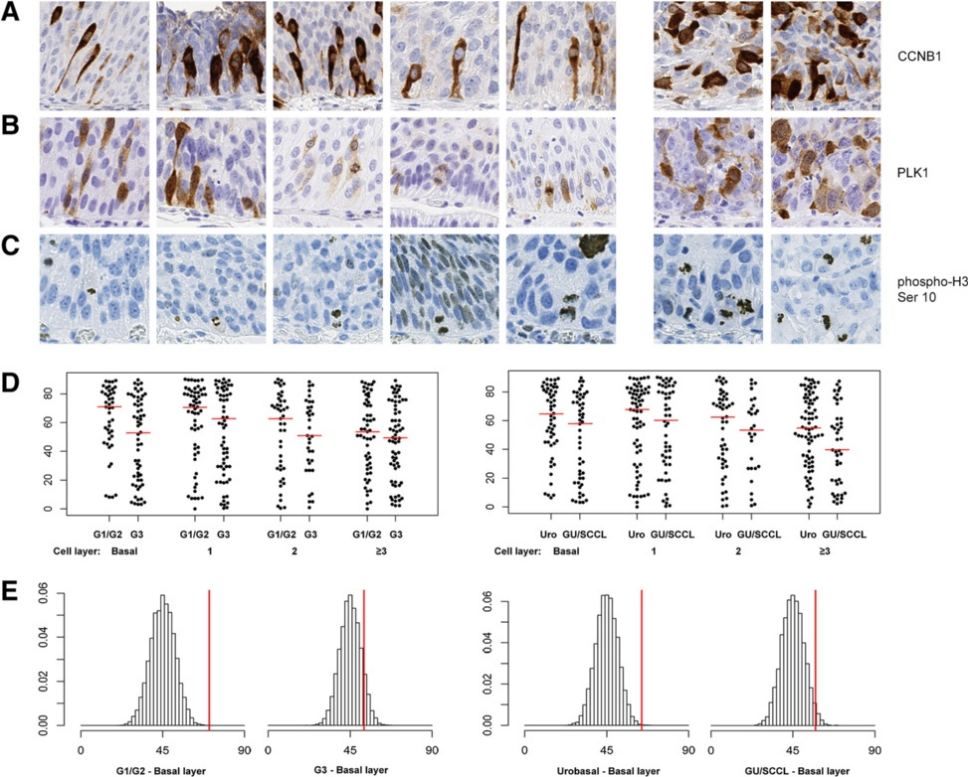
Cell divisions show preferential localization near the basal lamina and to close to 90 ° in low grade Urobasal tumors. **a** Immunohistochemical stainings of five Urobasal A cases (1–5) and two GU/SCCL cases (6–7) with anti-CCNB1. **b** Immunohistochemical stainings of five Urobasal A cases (1–5) and two GU/SCCL cases (6–7) with anti-PLK1. **c** Immuno-histochemical stainings of five Urobasal A cases (1–5) and two GU/SCCL cases (6–7) with antibodies for histone H3 phosphorylated at Ser10. **d** Bee-swarm dot-plots showing the distribution of division angles relative cell layer in low-grade vs. high-grade tumors and in Urobasal vs. GU/SCCL tumors. Red lines indicate median angle for each group. **e** Median observed basal cell division angles in low-grade vs. high-grade tumors and in Urobasal vs. SCCL tumors (red lines) plotted against the distribution of medians from 10^4^ randomly generated sets of angles of equal size

IHC analyses of CNNB1 expression in paraffin embedded material revealed a large proportion of G2/M cells located close to basal membranes. These observations were particularly evident in low grade Urobasal tumors. Furthermore, a large proportion of the CCNB1 positive cells were in contact with the basal membrane (Fig. 7a). By rescreening slides stained with antibodies for PLK1, also a G2/M specific marker, similar structures were observed as well as occasional cells with metaphase structures attached to the basal membrane (Fig. 7b). To obtain a more direct visualization of dividing cells we applied an antibody for phosphorylated histone H3 (Ser10) (Fig. 7c). The phospho-HISTH3 antibody confirmed that cell divisions were localized to predominantly basal and suprabasal cell layers and frequently were in contact with the basal membrane in UroA cases. We next identified 416 phospho-HISTH3 positive mitotic structures and measured the division angle relative to the nearest basal membrane. This investigation showed enrichment for division angles close to 90° when in close proximity to the basal membrane in low grade and UroA tumors (Fig. 7d-e). Thus, the distribution of cell division angles suggest that basal asymmetric cell divisions occur preferentially in UroA and low grade tumors, a pattern reminiscent of the regulated proliferation of normal urothelium.

## Discussion

The RXRA receptor protein forms heterodimers with the peroxisome proliferator-activated receptor-gamma (PPARG) and with retinoic acid receptors (RARs). Activation of PPARG and RAR both induce urothelial differentiation and expression of terminal differentiation genes [36, 37]. Knockdown of GATA4 and GATA6 abrogates the induction of UPK genes, showing that the GATA family of transcription factors acts as mediators of the RAR/RXRA induced response [36]. In addition, GATA4 and GATA6 as well as GATA2 and GATA3 show RXRA and PPARG ChIP-Seq binding sites in their promoters. PPARG/RXRA and RAR/RXRA signaling ultimately leads to the induction of the regulatory factor FOXA1 [36, 23]. Hence, the RAR/PPARG/RXRA FOXA1/GATA circuit shows strong involvement in differentiation of the normal urothelium. Our findings show that PPARG, RXRA, FOXA1 and GATA3 were strongly downregulated in the SCC-like tumors. Although RAR expression did not vary between subtypes, RA signaling may still be impaired as ALDH1A2 was downregulated and CYP26B1 was upregulated in the SCCL subtype. Additional downregulated factors potentially activated by the PPARG/RXRA/RAR system included *ELF3*, *TBX2* and *TBX3* [22, 38, 39]. Methylation of *TBX2* and *TBX3* has been found to be associated with urothelial tumor progression [40], however their full role in urothelial carcinoma is not yet clear. PPARG regulates the balance between glucose and lipid oxidation [41] and drives differentiation through a metabolic shift to lipid metabolism in prostate epithelia [42]. A key player in this process may be the transcription/nuclear factor ADIRF. Little is known about this factor except that it acts upstream of PPARG in lipid metabolism regulation [26]. Hence, downregulation of PPARG and ADIRF may contribute to an undifferentiated basal cell state by inhibiting lipid metabolism. STAT3 showed increased protein expression and phosphorylation in SCCL tumors. The importance of STAT3 in transforming normal urothelium to carcinoma in situ has been elegantly shown in transgenic mouse models [43]. The keratinizing squamous phenotype that characterizes the SCCL subtype may be a consequence of altered PPARG levels, as coordinated downregulation of PPARG and PTEN induces squamous differentiation of urothelium [44]. Conversely, activation of PPARG and inactivation of EGFR signaling reverses squamous metaplasia and induces urothelial differentiation [45]. Hence, downregulation of PPARG/RXRA and high EGFR expression combined with phospho-activation of STAT3 in SCCL UC most likely contribute to a basal/SCC-like phenotype and suggest that the SCCL subtype has a block in the normal differentiation of the urothelium.

Our analyses highlighted *FOXM1* and members of the *E2F* family to have key roles in determining the GU and SCCL phenotypes. The roles of E2F transcription factors and their interactions with RB related proteins in cell cycle regulation is well established and we have described a *RB1/E2F3* genomic circuit specifically operating in the GU subtype of tumors [6]. FOXM1 accumulates during the cell cycle but is kept in an inactive state by autorepression. At G2 FOXM1 is relieved from repression by CCNA/CDK phosphorylation and a positive feedback loop with PLK1 ensures hyperactivation of FOXM1 that ultimately triggers the G2 to M transition [31]. Our data show that FOXM1 and PLK1 protein expression levels are strongly associated in UC and that the PLK1-FOXM1 axis is activated in GU and SCCL cases. Activated FOXM1 binds to the DREAM transcription factor complex necessary for completion of the cell cycle [46]. Hence, the colocalization of FOXM1 ChIP-Seq binding sites with ChIP-Seq binding sites for members of the DREAM transcription factor complex in the identified gene signature strongly suggests that FOXM1 functions as a key regulator of cell division in the GU and SCCL subtypes. FOXM1 also influences cellular differentiation. Loss of *FOXM1* expression in mammary glands leads to an increase in the number of differentiated cells whereas *FOXM1* overexpression results in expansion of undifferentiated cells, suggesting that *FOXM1* expressing cells fail to exit the progenitor cell pool and to differentiate properly [47]. *FOXM1* is also upregulated during regeneration in response to injury and may be required for the expansion of regenerative stem/progenitor cells [48]. In addition, *FOXM1* overexpression has been linked with tumor aggressiveness [49] and with the accumulation of genomic alterations [50]. This latter finding is in accordance with the more complex genomes seen in GU and SCCL tumors [6]. Taken together, our data suggest the PLK1-FOXM1 axis as a key player in urothelial carcinoma biology by regulating a dynamic switch from a differentiated non-proliferative state to a proliferative undifferentiated state. This suggests that differentiation in GU is bypassed, not blocked, explaining why advanced high grade GU tumors frequently express differentiation markers, albeit in an aberrant way [4, 51].

UroA tumors showed maintained PPARG, RXRA, FOXA1 and GATA3 protein expression in the majority of cases. This likely contributes to the differentiated phenotype seen in this subtype. The link between PPARG expression and UroA, *i.e.*, low-grade stage Ta tumors, has also been suggested by Biton *et al*. [52]. Even though GU tumors frequently express subsets of the same proteins, GU tumors rarely express the complete set. This impairment may be linked to elevated *FOXM1* expression as FOXM1 is known to abrogate GATA3 activity [47] and GATA3 was the most frequently downregulated protein of this set of proteins in GU. We noted sharp shifts of positive to negative *FABP4*/*CRABP2* and *FABP5*/*CRABP2* gene expression ratios in GU cases. The ratio of *FABP5* and *CRABP2* expression is crucial for fatty acid ligand shuttling and ultimately affects cell fate [33, 34]. Hence, even if GU cases may express the luminal associated PPARG [53], this protein is expressed in a completely different context in GU compared with UroA.

In experimental systems undifferentiated cells express the posterior *HOXA* genes while RA differentiated cells express the anterior *HOXA* genes [54] creating an “anterior” signature in differentiated and a “posterior” signature in undifferentiated cells [55]. In our data the anterior signature was prevalent in UroA, the subtype with the most explicit signs of differentiation *i.e.*, low pathological grade, largely maintained urothelial like histology and occasional luminal expression of UPKs [4]. Furthermore, anterior *HOXA* and *HOXB* gene promoters have abundant PPARG, RXRA, FOXA1, GATA3, as well as with RARA protein binding sites. Hence, we propose a gene regulatory link between the PPARG/RXRA and FOXA1/GATA3 systems and anterior *HOXA* and *HOXB* expression. Using ChIP-Seq data for mouse HOXA2 we showed that several genes important for urothelial differentiation, upregulated in UroA, were potentially HOXA2 regulated. These genes included the transcription factors *GATA3*, *ELF3* and *TP63* required for urothelial stratification [56], and members of the TGFB signaling pathway. Hence, the presented data delineates a differentiation program involving PPARG/RXRA as well as anterior *HOXA* and *HOXB* genes that are, at least partly, operating in UroA, that has deteriorated in GU and is downregulated in SCCL tumors.

We [4], and others [7, 53, 57], have noted the analogy between the urothelial carcinoma SCCL subtype and the breast cancer basal-like subtype. Apart from the fact that the role of the PPARG/RXRA heterodimer in urothelial cancer is exchanged with the ESR1 hormone receptor in breast cancer, the key transcription factors FOXA1 and GATA3 are downregulated in the basal/SCC-like subtypes of both tumor types. Furthermore, there is ample evidence that both FOXA1 and GATA3 have key roles in luminal epithelial mammary differentiation [58, 59]. Altered lipid metabolism seems to be a key feature in establishing the SCCL UC and basal-like BC subtypes. The role of the regulatory factor ADIRF in this process, however, remains to be established. The similarities also included breast cancer basal-like upregulated genes which were, as in UC, enriched for STAT3 binding in their promoters. The analogy could also be extended to the lung squamous cell carcinoma subtype and suggests that downregulation of FOXA1 and GATA3 as well as upregulation/activation of EGFR and STAT3 as fundamental components of a generalized basal/squamous-like tumor phenotype. Although to a lesser extent, the similarities between UC and BC subtypes may be extended to include the Genomically Unstable and Urobasal subtypes as well. In this respect Genomically Unstable would be analogous to the Luminal B and HER2-enriched subtypes characterized by being aggressive, undifferentiated and PLK1-FOXM1 driven and the Urobasal subtype analogous to Luminal A as both show dependence of nuclear hormone receptors for their phenotypes. Thus, tumor types as disparate as bladder and breast cancer may be determined by analogous underlying regulatory systems even though the resulting phenotype and tissue of origin may differ.

## Conclusions

**Fig. 8:**
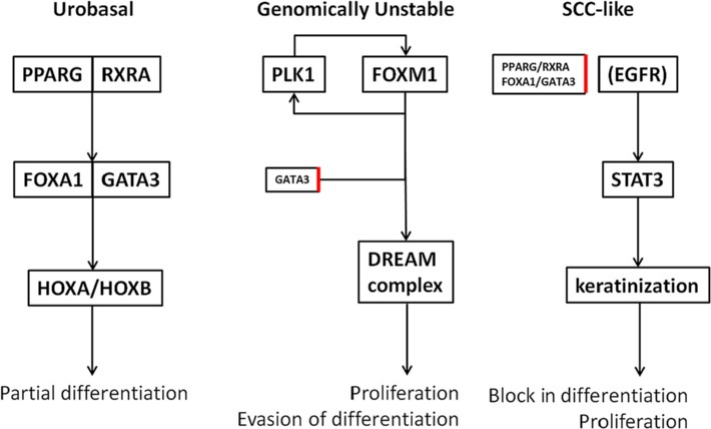
Regulatory systems operating in UC subtypes. Schematic summary of the gene regulatory systems operating in urothelial carcinoma subtypes, as suggested by the present findings

The emerging picture is that Urobasal, Genomically Unstable and SCC-like subtypes of urothelial carcinoma represent three fundamentally different tumor types (summarized in Fig. 8). Urobasal tumors maintain the suggested urothelial differentiation axis composed of PPARG/RXRA, FOXA1/GATA3 and HOXA/HOXB genes more or less intact. Furthermore, our data indicate that a large proportion of cell divisions in low grade Urobasal tumors are associated with the basal membrane, possibly giving rise to asymmetrical divisions. A critical difference between the Urobasal and the Genomically Unstable and SCC-like tumors, is an increase of proliferative activity independent of a basal membrane. The elevated proliferative activity was observed as a strong signal for PLK1-FOXM1 regulated genes in both subtypes. However, whereas SCC-like tumors seem to have a block in urothelial differentiation, the Genomically Unstable tumors appear to evade differentiation in a more dynamic manner, potentially through the action of FOXM1. Core components of the described regulatory systems appeared to operate across multiple tumor types such as breast and lung cancer. Even though the presented exploratory data is suggestive, the assumptions have to be validated in experimental systems. Irrespectively, our data point to major differences at the gene regulatory level between urothelial carcinoma subtypes.

## Additional files

Additional file 1: Table S4. Gene lists from SAM and QTC analyses. Additional file 2:
**Figure S1.** Pathways indicated in urothelial differentiation. **Figure S2.** Promoter binding overlap in the SCCL downregulated genes. **Figure S3.** Genes involved in retinoic acid synthesis and degradation. **Figure S4.** Identification of the SCCL subtype in the TCGA bladder cancer dataset. **Figure S5.** Transcription factors involved in urothelial differentiation are retained in Urobasal A tumors and partially lost in the Genomically Unstable and SCCL tumors. **Figure S6.** Sample ranking by HOX switch score. **Figure S7.** The HOXA/HOXB switch in urothelial and breast cancer**. Table S1.** Transcription factor binding enrichment in promoters of downregulated genes of the SCCL subtypes in bladder and basal-like subtype in breast. **Table S2.** Ranking of transcription factors within the list of downregulated genes in the SCCL and basal-like subtypes. **Table S3.** Transcription Factor binding enrichment in the Late Cell Cycle QTC.

## Abbreviations

BC: Breast cancer
ChIP-Seq: Chromatin Immunoprecipitation Sequencing
GU: Genomically unstable
GO: Gene ontology
QTC: Quality threshold clustering
RA: Retinoic acid
SCCL: Squamous cell carcinoma-like
SAM: Significance analysis of microarrays
TCGA: The cancer genome atlas
TSS: Transcription start site
UC: Urothelial carcinoma
