# Supplementary material for: Molecular subtypes of urothelial carcinoma are defined by specific gene regulatory systems
Source: BMC Med Genomics. 2015 May 26;8:25. doi: 10.1186/s12920-015-0101-5 (PMC4446831; doi:10.1186/s12920-015-0101-5)
Supplement: Additional file 2: — Figure S1. Pathways indicated in urothelial differentiation. Figure S2. Promoter binding overlap in the SCCL downregulated genes. Figure S3. Genes involved in retinoic acid synthesis and degradation. Figure S4. Identification of the SCCL subtype in the TCGA bladder cancer dataset. Figure S5. Transcription factors involved in urothelial differentiation are retained in Urobasal A tumors and partially lost in the Genomically Unstable and SCCL tumors. Figure S6. Sample ranking by HOX switch score. Figure S7. The HOXA/HOXB switch in urothelial and breast cancer. Table S1. Transcription factor binding enrichment in promoters of downregulated genes of the SCCL subtypes in bladder and basal-like subtype in breast. Table S2. Ranking of transcription factors within the list of downregulated genes in the SCCL and basal-like subtypes. Table S3. Transcription Factor binding enrichment in the Late Cell Cycle QTC. [file 12920_2015_101_MOESM2_ESM.pdf]

Molecular subtypes of urothelial carcinoma are defined by specific gene regulatory systems

Pontus Eriksson[1]
Email: pontus.eriksson@med.lu.se
Mattias Aine[1]
Email: mattias.aine@med.lu.se
Srinivas Veerla[1]
Email: srinivas.veerla@med.lu.se
Fredrik Liedberg[2]
Email: Fredrik.liedberg@med.lu.se
Gottfrid Sjödahl[2]
Email: Gottfrid.sjodahl@med.lu.se
Mattias Höglund[1,*]
*Corresponding author
Email: mattias.hoglund@med.lu.se

[1] Division of Oncology and Pathology, Department of Clinical Sciences Lund, Lund University, Lund, Skåne, SE-223 81, Sweden.
[2] Division of Urological Research, Department of Clinical Sciences Malmö, Lund University, Malmö, Skåne, SE-205 02, Sweden.

TABLE OF CONTENTS

SUPPLEMENTAL FIGURES

Figure S1. Pathways indicated in urothelial differentiation.

Figure S2. Promoter binding overlap in the SCC-like downregulated genes.

Figure S3. Genes involved in retinoic acid synthesis and degradation.

Figure S4. Identification of the SCC-like subtype in the TCGA bladder cancer dataset.

Figure S5. Transcription factors involved in urothelial differentiation are retained in Urobasal A tumors and partially lost in the Genomically Unstable and SCC-like tumors.

Figure S6. Sample ranking by HOX switch score.

Figure S7. The HOXA/HOXB switch in urothelial and breast cancer.

SUPPLEMENTAL TABLES

Table S1. Transcription factor binding enrichment in promoters of downregulated genes of the SCC-like subtypes in bladder, and basal-like subtype in breast.

Table S2. Ranking of transcription factors within the list of downregulated genes in the SCC-like and basal-like subtypes.

Table S3. Transcription Factor binding enrichment in the Late Cell Cycle QTC.

Table S4. Gene lists from SAM and QTC analyses. (Additional File 1)

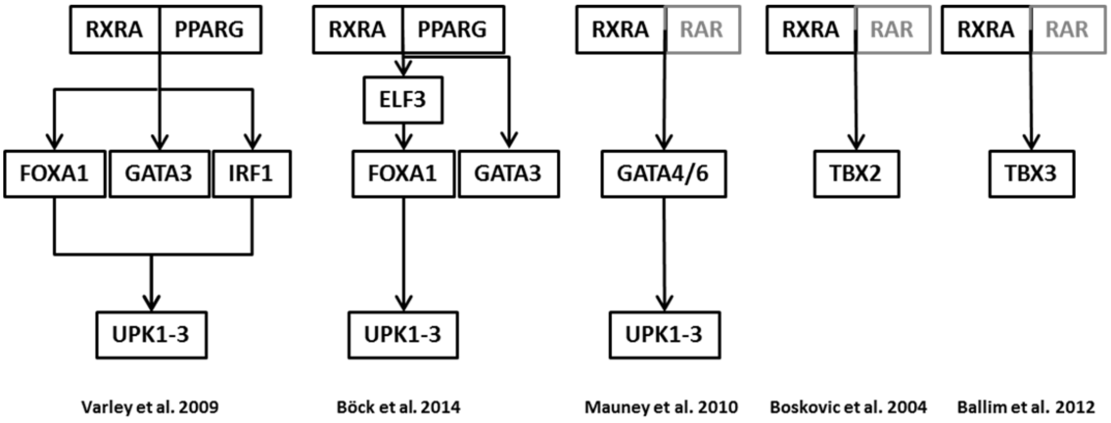

Figure S1. Pathways indicated in urothelial differentiation.
Varley and co-workers [23] examined which intermediary factors that act downstream of PPARG, as uroplakins and cytokeratin expression are late events following PPARG activation in NHU cell cultures and does not have any strong peroxisome proliferator response element (PPRE) binding sites for the PPARG-RXRA complex itself. IRF-1 and FOXA1 does contain PPRE binding sites, and their expression was rapidly induced through simultaneous PPARG activation and EGFR inhibition. siRNA knockdown of either FOXA1 or IRF-1 abrogated the PPARG-induced induction of multiple late urothelial differentiation-associated genes. They also find GATA3 upregulated by the simultaneous activation and inhibition of PPARG and EGFR respectively, although at later time point. **Böck** and co-workers [22] examined the expression-changes occurring during induced differentiation of cultured NHU by two separate methodologies in two separate cell cultures derived from two non-cancerous human urothelial tissue samples, and found that ELF3 was one of the shared transcription factors induced by both differentiation strategies. ELF3 knockdown resulted in reduced expression of FOXA1 and GRHL3 in response to induced differentiation through PPARG activation. Pretreatment with the PPARG antagonist T0070907 prior to PPARG activation blocked ELF3 mRNA and protein induction, confirming that ELF3 is a downstream target of PPARG. GATA3 was found among the top 1000 genes overexpressed by both differentiation strategies. **Mauney** and co-workers [36] used all-trans retinoic acid (RA) to stimulate ESC which led to downregulation of the pluripotency factor OCT-5 and a strong upregulation of UP1A, UP1B, UP2, UP3A, and UP3B. The expression of the characteristic uroplakins was found to be dependent on GATA4 and GATA6, also upregulated by RA stimulation, highlighting the importance of GATA factors for the ESC specification towards an urothelial lineage. **Boskovic and Niles** [39] showed that TBX2 is a direct target of retinoic acid signaling. An increase in TBX2 mRNA was seen within 2 hours after RA stimulation, indicating that no new protein synthesis was required for TBX2 induction. They identified retinoic acid response elements (RARE) in the promoter region of TBX2, and showed that these were functional using a luciferase assay vector with the TBX2 promoter sequence. Mutating the RARE sites reduced or abrogated the ability to respond to RA. The TBX2 promoter has ChIP-Seq binding sites for GATA3 and FOXA1 according to ENCODE/CISTROME data, and for RXRA, PPARG, FOXA1, and RARA according to Kittler data. **Ballim** and co-workers [38] provided evidence that TBX3 is a direct RAR-RXR target by co-transfecting cells with a RAR and RXR expressing vector along with a luciferase vector containing the TBX3 promoter. Addition of RA to the cell culture resulted in a significant increase in luciferase reporter activity. Retinoic acid response elements were also found within the TBX3 promoter sequence, which when mutated lowered the response to RA. The TBX3 promoter has ChIP-Seq binding sites for GATA3, FOXA1, PPARG according to ENCODE/CISTROME data, and for RXRA, FOXA1, and RARA according to Kittler data. While the specific role of the TBX genes in bladder cancer is yet unclear, methylation of both TBX2 and TBX3 was found to be associated with tumor progression in bladder cancer [40].

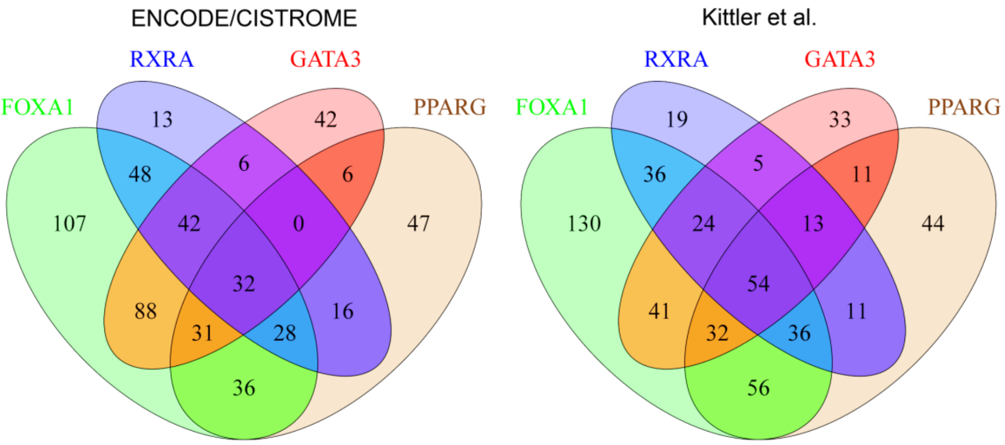

Figure S2. Promoter binding overlap in the SCC-like downregulated genes.
Co-occurrence of FOXA1, RXRA, GATA3 and PPARG binding in the promoter regions of the 829 SAM significant genes downregulated in the SCC-like subtype. ENCODE/CISTROME dataset (left), and the Kittler dataset (right).

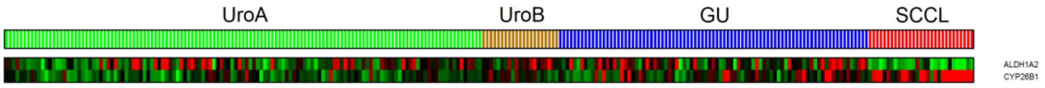

Figure S3. Genes involved in retinoic acid synthesis and degradation.
Expression levels of ALDH1A2 and CYP26B1 involved in retinoic acid synthesis and degradation is altered in the SCC-like subtype.

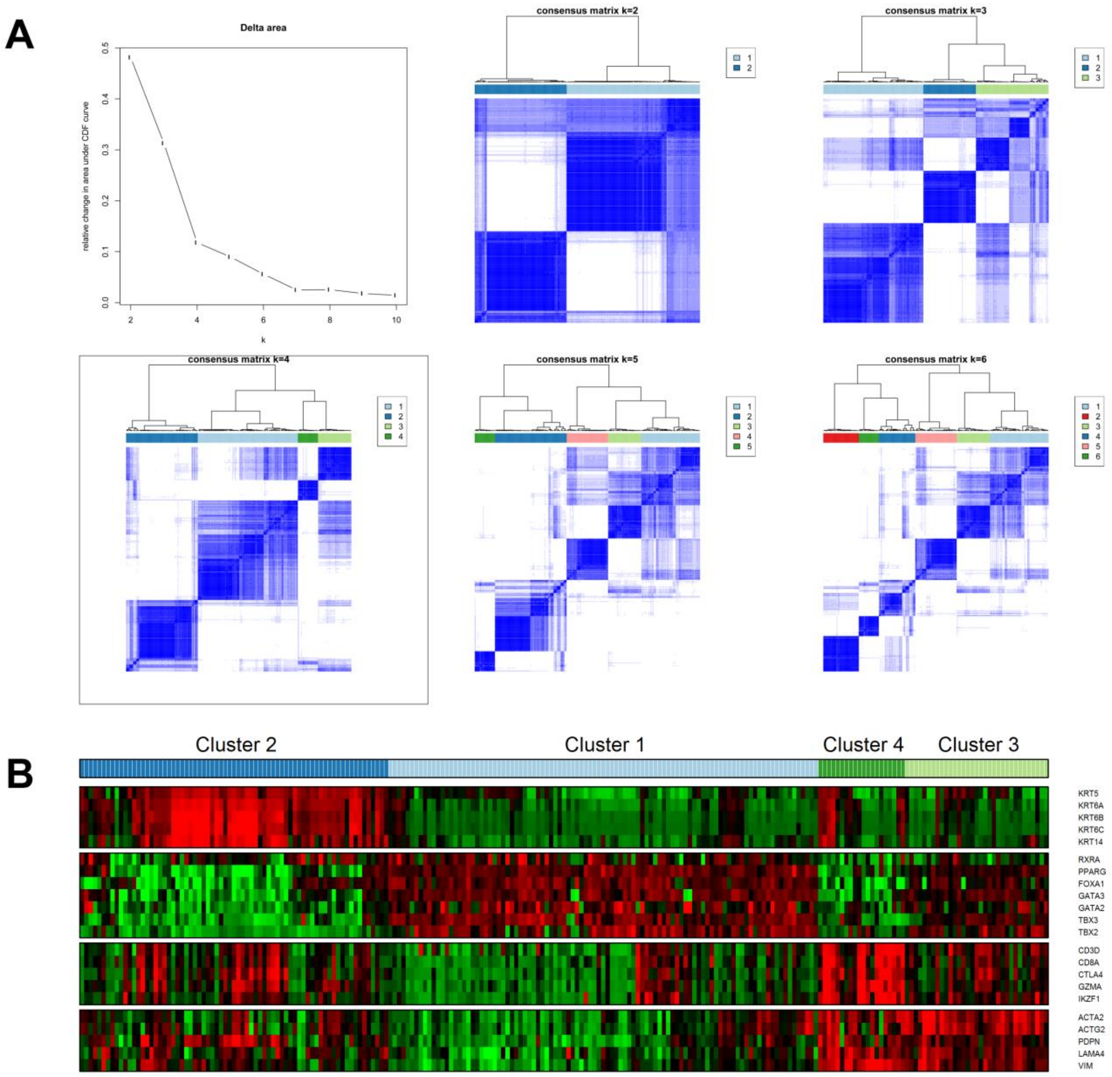

Figure S4. Identification of the SCC-like subtype in the TCGA bladder cancer dataset.

(**A**) Gene expression data (RNASeqV2) from 242 samples was downloaded from the TCGA bladder urothelial carcinoma data portal. The dataset included 223 MI tumor samples and 19 “normal urothelial samples”. Rsem estimate values for each sample was multiplied with 1 000 000 and an offset of 1 was added to each value. The data was log2 transformed, and the 19 normal samples were removed from the data matrix. The standard deviation for each gene was calculated, and the genes with a SD>90th percentile (2053 genes) were used to cluster the samples using the ConsensusClusterPlus package in R [24]. ConsensusClusterPlus settings were; maxK=10, reps=1000, pItem=0.8, pFeature=1, clusterAlg="hc", innerLinkage="ward", finalLinkage="ward", distance="pearson". A four cluster solution was chosen. (**B**) Cluster 2 contained tumors of the SCC-like subtype, identified by their keratinization and loss of urothelial differentiation transcription factors, while Cluster 3 and 4 contained tumors with a high level of inflammation and ECM/myofibroblast content. Cluster 1 was composed of Genomic Unstable or Urobasal tumors. Signatures: Keratins: KRT5, KRT6A, KRT6B, KRT6C, KRT14; Urothelial Differentiation: RXRA, PPARG, FOXA1, GATA3, GATA2, TBX3, TBX2. T-cell markers of infiltration: CD3D, CD8A, CTLA4, GZMA, IKZF1; ECM and myofibroblasts: ACTA2, ACTG2, PDPN, LAMA4, VIM.

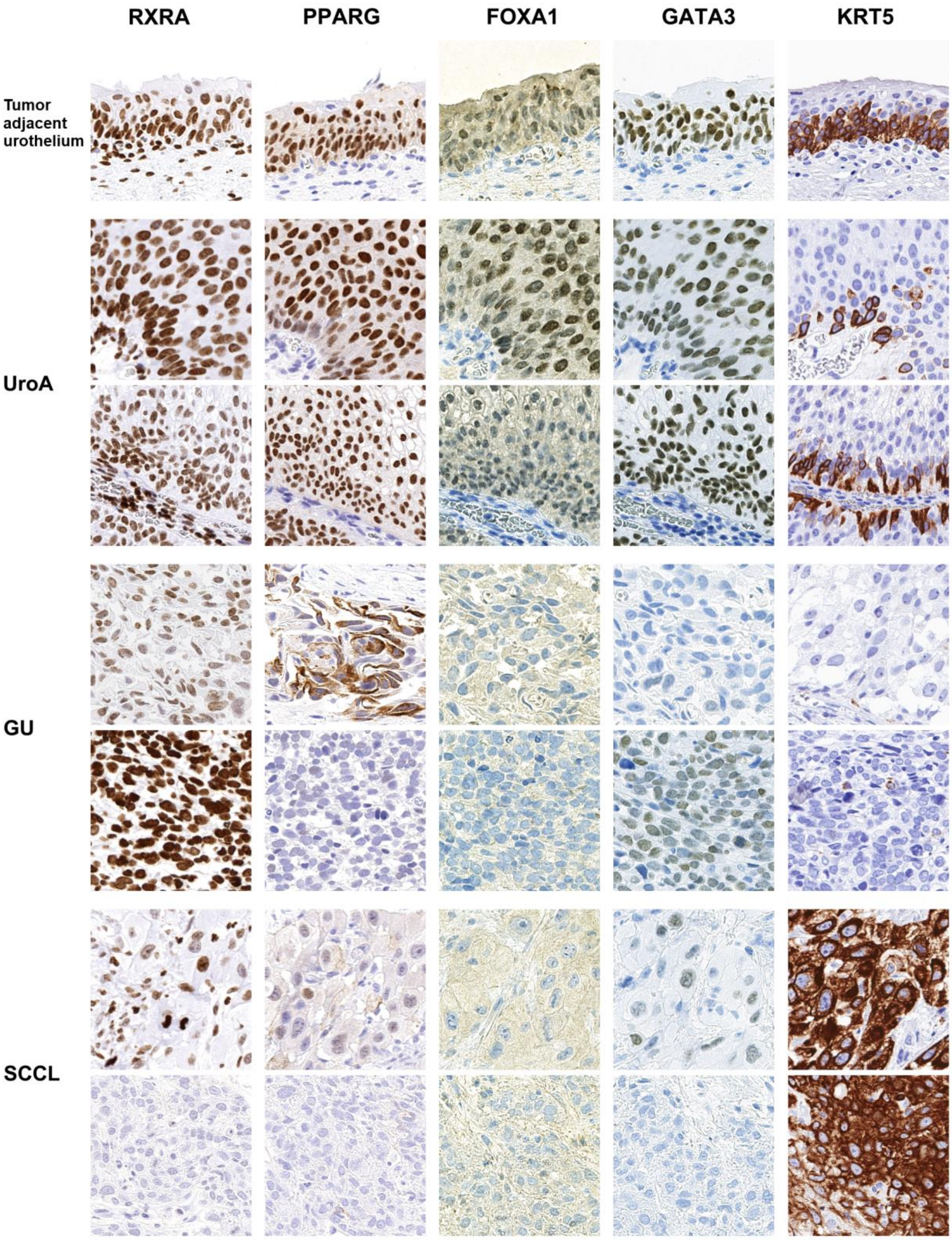

Figure S5. Transcription factors involved in urothelial differentiation are retained in Urobasal A tumors and partially lost in the Genomically Unstable and SCC-like tumors.
Tumor adjacent urothelium show nuclear staining of RXRA, PPARG, FOXA1, and GATA3. KRT5 indicate the basal cell population and the identity of SCC-like tumors.

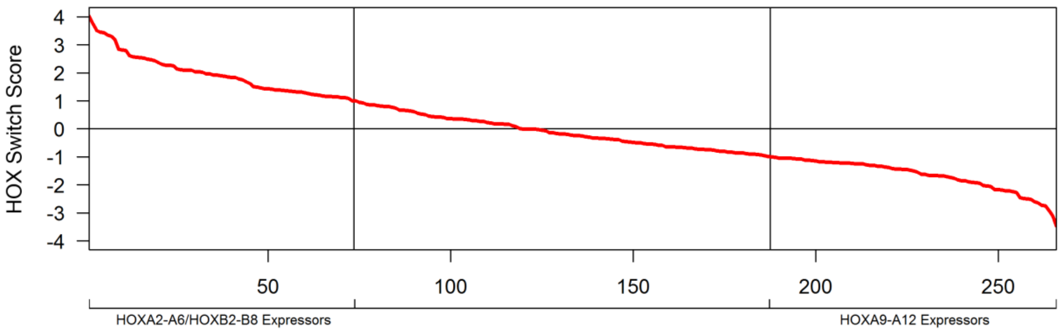

Figure S6. Sample ranking by HOX switch score.
The HOX switch score was calculated by adding the average expression of the early HOXA genes (HOXA2, HOXA3, HOXA4, HOXA5, and HOXA6) and the average expression of the HOXB genes (HOXB2, HOXB3, HOXB4, HOXB5, HOXB6, HOXB7, HOXB8) and subtracting the average expression values of the late HOXA genes (HOXA9, HOXA10, HOXA11, HOXA13). After sorting the samples on their HOX switch score, an arbitrary cutoff point of 1 and -1 was set to define early and late HOX expressers. This resulted in 73 samples being defined as HOXA2-A6/HOXB2-B8 expressers, 114 as intermediate, and 79 samples as HOXA9-A12 expressers. A SAM analysis between the HOXA2-A6/HOXB2-B8 expressers and the HOXA9-A12 expressers resulted in 672 upregulated genes.

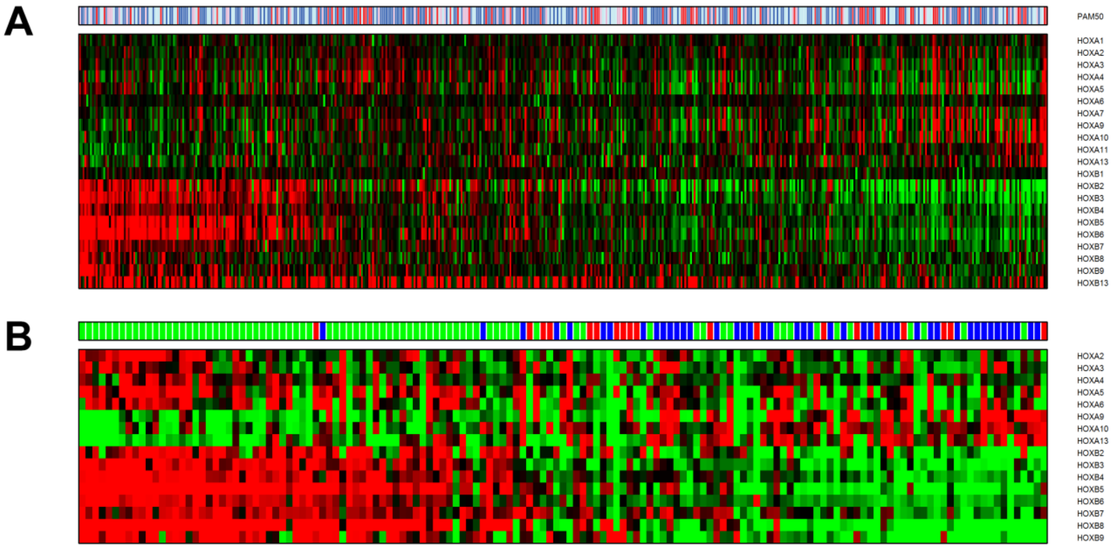

Figure S7. The HOXA/HOXB switch in urothelial and breast cancer.
The HOXA/HOXB switch is present in the Chungbuk bladder cancer dataset [8] (**A**) but not in the TCGA breast cancer dataset [10] (**B**). Tumors organized according to HOXA/HOXB switch score in each panel.

A

Lund data. SCCL down

Factor	Data set	p-value*	%-presence†
RXRA	Encode	$6x10^{-7}$	24
PPARG	Cistrome	$8x10^{-4}$	25
FOXA1	Encode	$3x10^{-17}$	53
GATA3	Encode	$2x10^{-13}$	34
RXRA	Kittler	$2x10^{-7}$	25
PPARG	Kittler	$2x10^{-11}$	33
FOXA1	Kittler	$2x10^{-18}$	53
GATA3	Kittler	$3x10^{-9}$	28

B

TCGA Bladder data. SCCL down

Factor	Data set	p-value	%-presence
RXRA	Encode	$3x10^{-6}$	21
PPARG	Cistrome	$4x10^{-4}$	24
FOXA1	Encode	$2x10^{-13}$	47
GATA3	Encode	$3x10^{-6}$	25
RXRA	Kittler	$5x10^{-19}$	26
PPARG	Kittler	$2x10^{-11}$	29
FOXA1	Kittler	$4x10^{-23}$	49
GATA3	Kittler	$9x10^{-7}$	23

C

TCGA Breast data. Basal-like down

Factor	Data set	p-value	%-presence
RXRA	Encode	$2x10^{-8}$	21
PPARG	Cistrome	$1x10^{-12}$	26
FOXA1	Encode	$8x10^{-35}$	50
GATA3	Encode	$2x10^{-29}$	30
RXRA	Kittler	$2x10^{-18}$	25
PPARG	Kittler	$2x10^{-17}$	29
FOXA1	Kittler	$8x10^{-37}$	49
GATA3	Kittler	$8x10^{-22}$	26
ESR1	Kittler	$2x10^{-35}$	34
ESR1	Encode	$4x10^{-19}$	17

D

TCGA data. SCCL/basal-like down overlap

Factor	Data set	p-value	%-presence
RXRA	Encode	$3x10^{-7}$	25
PPARG	Cistrome	$5x10^{-4}$	26
FOXA1	Encode	$6x10^{-10}$	50
GATA3	Encode	$2x10^{-13}$	33
RXRA	Kittler	$1x10^{-8}$	27
PPARG	Kittler	$2x10^{-6}$	31
FOXA1	Kittler	$4x10^{-15}$	53
GATA3	Kittler	$1x10^{-7}$	27
ESR1	Kittler	$5x10^{-14}$	37
ESR1	Encode	$9x10^{-10}$	20

Table S1. Transcription factor binding enrichment in promoters of downregulated genes of the SCC-like subtypes in bladder, and basal-like subtype in breast.

In-silico binding enrichment of transcription factors in the genes downregulated in the (**A**) SCC-like subtype in the Lund data, (**B**) SCC-like subtype in the TCGA bladder cancer dataset, (**C**) basal-like subtype in the TCGA breast cancer dataset, and (**D**) the overlap of downregulated genes of the SCC-like and basal-like subtypes in the TCGA datasets.

* p-values were calculated using a one-tailed Fisher's exact test on the number of promoters with binding in a given gene list.

† %-presence indicates the percentage of genes in each list that had binding sites.

	Lund data SCCL down (TF)	Lund data SCCL down (829)	TCGA Bladder SCCL down (TF)	TCGA Bladder SCCL down (2054)	TCGA Breast basal-like down (TF)	TCGA Breast basal-like down (3638)
ADIRF	1	2	4	24	20	139
GATA3	2	8	7	73	5	11
FOXA1	3	10	12	120	1	1
TBX3	4	16	1	6	18	303
PPARG	5	22	3	12	NS	NS
TBX2	8	44	2	10	NS	NS
ELF3	11	80	30	286	NS	NS
GATA2	16	131	16	165	34	608
RXRA	38	324	NS	NS	NS	NS
ESR1	NS*	NS	NS	NS	6	13

Table S2. Ranking of transcription factors within the list of downregulated genes in the SCC-like and basal-like subtypes.
Column 1, 3, and 5 show the respective rank among downregulated transcription factors, while column 2, 4, and 6 show the rank in the full list of downregulated genes. The full list of downregulated genes is provided as a supplemental excel file (Additional File 1: Table S4).
* NS indicates that the gene was not significantly downregulated.

Lund dataset. Late Cell Cycle QTC

Factor	Data set	p-value*	%-presence†
HDAC1	Encode	$3x10^{-42}$	91
SAP30	Encode	$6x10^{-36}$	86
FOXM1	Encode	$5x10^{-35}$	76
E2F4	Encode	$6x10^{-35}$	95
CHD2	Encode	$9x10^{-32}$	98
MYBL2	Encode	$4x10^{-31}$	75
KDM5B	Encode	$5x10^{-31}$	91
CREB1	Encode	$4x10^{-30}$	90
NFYB	Encode	$7x10^{-30}$	75
E2F1	Encode	$8x10^{-30}$	94
NFYA	Encode	$3x10^{-29}$	67
SIN3AK20	Encode	$4x10^{-28}$	98
SIN3A	Encode	$9x10^{-28}$	95
PHF8	Encode	$2x10^{-27}$	94
IRF1	Encode	$7x10^{-27}$	89
PML	Encode	$9x10^{-26}$	87
VDR	Cistrome	$9x10^{-23}$	90
SP1	Encode	$9x10^{-23}$	90
E2F6	Encode	$3x10^{-22}$	90
ELF1	Encode	$9x10^{-22}$	94

Table S3. Transcription Factor binding enrichment in the Late Cell Cycle QTC.
In-silico ChIP-Seq enrichment of the Late Cell Cycle QTC.
* p-values were calculated using a one-tailed Fisher's exact test on the number of promoters with binding in a given gene list.
† %-presence indicates the percentage of genes in each list that had binding sites.

Table S4.
Gene lists from SAM and QTC analyses.

Sheet 1: SAM two class unpaired between SCC-like bladder tumors VS rest. Lund dataset.

Sheet 2: SAM two class unpaired between TCGA bladder SCCL-like tumors (Cluster 2) VS Genomic Unstable/Urobasal tumors (Cluster 1).

Sheet 3: SAM two class unpaired between TCGA breast basal-like tumors VS rest.

Sheet 4: SAM two class unpaired between HOXA2-A6/HOXB2-B8 expressers vs HOXA9-A12 expressers (Filtered from HOX genes involved in defining the groups).

Sheet 5: HOXA2-bound genes among the upregulated genes of the HOXA2-A6/HOXB2-B8 expressers.

Sheet 6: QTC gene lists.
